# Heterogeneous nucleation of β-type precipitates on nanoscale Zr-rich particles in a Mg-6Zn-0.5Cu-0.6Zr alloy

**DOI:** 10.1186/1556-276X-7-300

**Published:** 2012-06-08

**Authors:** Hongmei Zhu, Gang Sha, Jiangwen Liu, Hongwei Liu, Cuilan Wu, Chengping Luo, Zongwen Liu, Rongkun Zheng, Simon P Ringer

**Affiliations:** 1 School of Materials Science and Engineering, South China University of Technology, Guangzhou, 510640, China; 2 Australian Centre for Microscopy and Microanalysis, The University of Sydney, New South Wales, 2006, Australia; 3 School of Mechanical Engineering, University of South China, Hengyang, 421001, China; 4 Center of High Resolution Electron Microscopy, School of Materials Science and Engineering, Hunan University, Changsha, 410082, China

**Keywords:** Mg alloys, Zn-rich precipitates, nanoscale Zr-rich particles, heterogeneous nucleation, electron microscopy

## Abstract

Zirconium (Zr) is an important alloying element to Mg-Zn-based alloy system. In this paper, we report the formation of the β-type precipitates on the nanoscale Zr-rich particles in a Mg-6Zn-0.5Cu-0.6Zr alloy during ageing at 180°C. Scanning transmission electron microscopy examinations revealed that the nanoscale Zr-rich [0001]_α_ rods/laths are dominant in the Zr-rich core regions of the as-quenched sample after a solution treatment at 430°C. More significantly, these Zr-rich particles served as favourable sites for heterogeneous nucleation of the Zn-rich β-type phase during subsequent isothermal ageing at 180°C. This research provides a potential route to engineer precipitate microstructure for better strengthening effect in the Zr-containing Mg alloys.

## Background

Mg-Zn-based alloys have attracted considerable attention due to their pronounced age-hardening effect [1-5]. The key strengthening precipitates in this alloy system have been considered as two types of Zn-rich precipitates, the rod-like β_1_′ precipitates perpendicular to the (0001)_α_ plane and the plate-like β_2_′ precipitates parallel to the (0001)_α_ plane [1-5]. Hardening by precipitation of β-type precipitates is believed to be the main strengthening mechanism of Mg-Zn-based alloys [1].

Recently, a peak-aged Mg-6Zn-0.5Cu-0.6Zr cast alloy has been reported to possess excellent mechanical properties with an ultimate tensile strength of 266.3 MPa, a 0.2% proof yield strength of 185.6 MPa and an elongation of 16.7% [5]. Both the strength and ductility of the newly designed Mg-6Zn-0.5Cu-0.6Zr alloy are superior to those of the traditional Mg-6Zn-xCu-0.5Mn alloys [5,6]. Since Zr-rich particles may form after a solution treatment in Zr-containing Mg alloys [2,7,8], the present research aims to unveil the effect of these pre-existing nanoscale Zr-rich particles on the formation of the subsequent β-type precipitates of the Mg-6Zn-0.5Cu-0.6Zr alloy during age hardening.

## Methods

The alloy with a nominal composition of Mg-6Zn-0.5Cu-0.6Zr (wt.%) for this study was prepared by melting high-purity Mg and Zn with Mg-28.78 wt.% Cu and Mg-31.63 wt.% master alloys, in a steel crucible and by casting into a permanent mould under an Ar atmosphere. Samples sectioned from the ingot were solution-treated for 24 h at 430°C. To investigate the microstructural evolution of the Zr-rich and Zn-rich precipitates, the water-quenched samples were subsequently aged in an oil bath for 20 and 120 h at 180°C. Thin foil specimens for scanning transmission electron microscopy (STEM) and transmission electron microscopy (TEM) were prepared by a twin-jet electropolisher using a solution of 10.6 g LiCl, 22.32 g Mg(ClO_4_)_2_, 200 ml 2-butoxi-ethanol and 1,000 ml methanol at about −45°C and 70 V. The STEM study was conducted using a JEOL 2200FS microscope (JEOL Ltd., Tokyo, Japan) equipped with a high-angle annular dark field (HAADF) detector and a Bruker energy dispersive X-ray spectrometer (EDXS) detector (Bruker AXS, Karlsruhe, Germany). The conventional TEM analysis was carried out using a JEOL 3000F microscope equipped with an Oxford EDXS detector (Oxford Instruments, Oxfordshire, UK).

## Results and discussion

**Figure 1:**
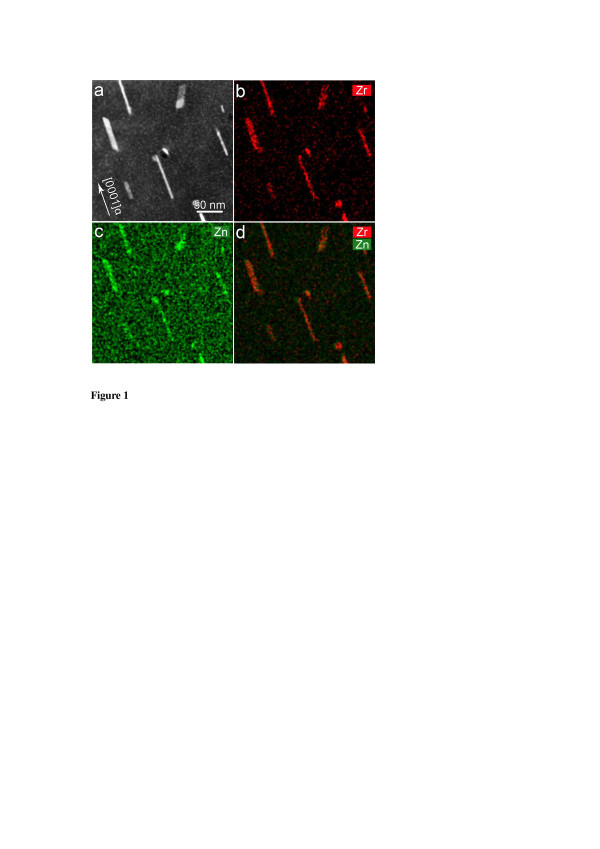
**Alloy quenched after a solution treatment at 430°C for 24 h.** The incident electron beam was parallel to ${\left[1\bar{2}10\right]}_{\alpha }$. **(a)** HAADF image, **(b)** Zr EDXS map, **(c)** Zn EDXS map and **(d)** a combined EDXS map of Zr and Zn.

Figure 1 shows the ${\left[1\bar{2}10\right]}_{\alpha }$ HAADF image and the corresponding EDXS map of the as-quenched sample after a solution treatment at 430°C for 24 h. Most particles in the Mg matrix are predominantly rods/laths elongated along the [0001]_α_ direction, with a length of 50 to approximately 200 nm, although a few particles are elongated along other directions. The rod/lath morphology of these particles was confirmed by further large-angle tilting experiments. The Zr map, Zn map and a combined Zr and Zn map, as shown in Figure 1b,c and d, reveal that all rod-like particles in bright contrast in Figure 1a are enriched with Zn and Zr. This is in good agreement with the previous reports showing that Zr-rich phases exist in various Zr-containing Mg-Zn-based alloys after a solution treatment [2,7,8]. EDXS analysis detected no enrichment of Cu in the Zr-rich particles.

**Figure 2:**
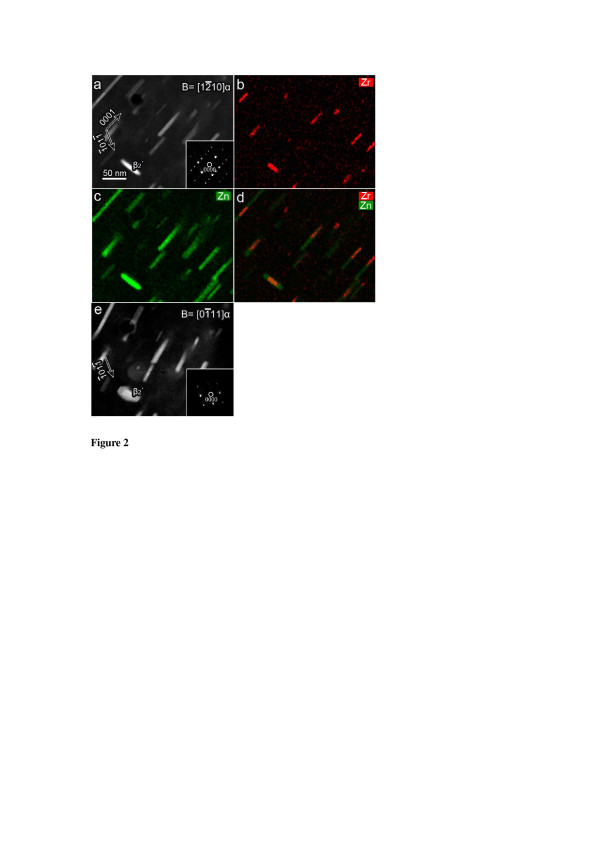
**Alloy aged at 180°C for 20 h.** The incident electron beam was parallel to ${\left[1\bar{2}10\right]}_{\alpha }$ in **(a-d)** and ${\left[0\bar{1}11\right]}_{\alpha }$ in **(e)**, respectively. (a) ${\left[1\bar{2}10\right]}_{\alpha }$ HAADF image, (b) Zr EDXS map, (c) Zn EDXS map, (d) a combined EDXS map of Zr and Zn and (e) ${\left[0\bar{1}11\right]}_{\alpha }$ HAADF image.

In order to investigate the effect of these pre-existing Zr-rich particles on the formation of Zn-rich strengthening precipitates during subsequent isothermal ageing, HAADF imaging and EDXS mapping were conducted on samples aged at 180°C for different time. The ${\left[1\bar{2}10\right]}_{\alpha }$ HAADF image of the 20-h-aged sample, as shown in Figure 2a, reveals that a dispersion of particles was mostly elongated along the [0001]_α_ direction, with only one marked β_2_′ perpendicular to the [0001]_α_ direction. After tilting a large angle of approximately 51° to the ${\left[0\bar{1}11\right]}_{\alpha }$ zone axis (Figure 2e), all particles observed in Figure 2a were found to be separate without overlapping with each other. The β_2_′ precipitate, marked in Figure 2a, is a plate containing a brighter core, which corresponds to an enrichment of Zr (Figure 2b). The Zr map, Zn map and a combined Zr and Zn map, as shown in Figure 2b,c, and d, demonstrate that most of the elongated particles were composites containing a Zn-rich part and a Zr-rich segment. Careful examinations of the EDXS maps and the HAADF image confirmed that each Zr-rich segment was located either at the end or in the middle of an individual elongated precipitate. Therefore, we conclude that those Zr-rich segments of the precipitates are, in fact, the remains of the Zr-rich particles initially present in the as-quenched condition. We further deduce that these Zr-rich particles served as a precursor phase for the heterogeneous nucleation of Zn-rich β_1_′ precipitates ([0001]_α_ rods) and β_2_′ precipitates ((0001)_α_ plates) in the Zr-rich core regions of the Mg alloy during subsequent ageing.

**Figure 3:**
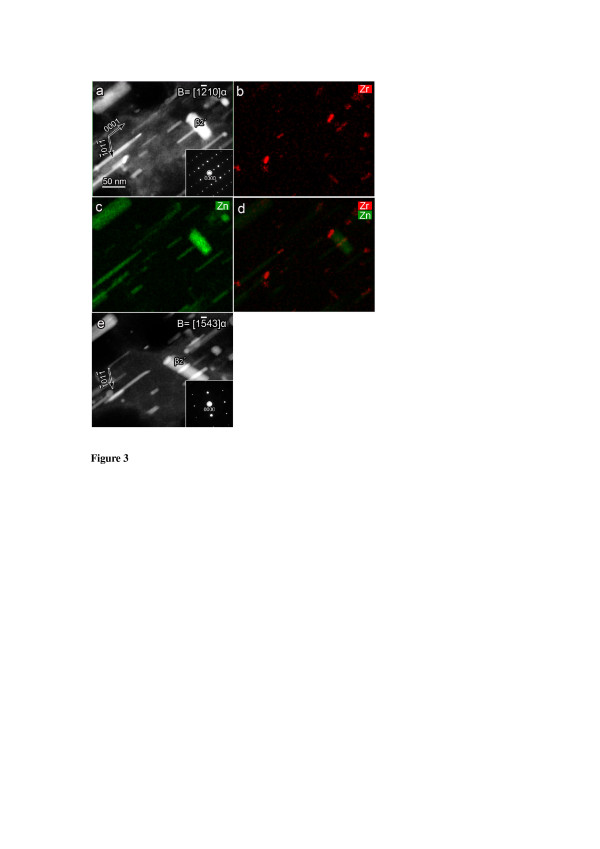
**Alloy aged at 180°C for 120 h.** The incident electron beam was parallel to ${\left[1\bar{2}10\right]}_{\alpha }$ in **(a-d)** and ${\left[1\bar{5}43\right]}_{\alpha }$ in **(e)**, respectively. (a) ${\left[1\bar{2}10\right]}_{\alpha }$ HAADF image, (b) Zr EDXS map, (c) Zn EDXS map, (d) a combined EDXS map of Zr and Zn and (e) ${\left[1\bar{5}43\right]}_{\alpha }$ HAADF image.

Figure 3 shows the HAADF image and the corresponding EDXS mapping result of the 120-h-aged sample. Both the length of [0001]_α_ β_1_′ rods and the thickness of (0001)_α_ β_2_′ plates grew significantly with the ageing time. The Zr map, Zn map and a combined Zr and Zn map, as shown in Figure 3b,c and d, indicate that many β_1_′ rods and the β_2_′ plate contain a Zr-rich segment. The sizes of Zr-rich segments observed in the 120-h-aged sample are smaller than those observed in the 20-h-aged sample. It appears that the size of the Zn-rich segments gradually increased at the expense of the Zr-rich segments during the isothermal ageing. After tilting approximately 36° from the ${\left[1\bar{2}10\right]}_{\alpha }$ beam direction, a ${\left[1\bar{5}43\right]}_{\alpha }$ HAADF image (Figure 3e) further confirms the existence of the Zr-rich segments in the Zn-rich precipitates. All experimental evidences above indicate that the heterogeneous nucleation on the pre-existing Zr-rich particles is significantly important for the formation of Zn-rich precipitates (β_1_′ and β_2_′) in the Zr-rich core regions of the Mg alloy during ageing at 180°C.

**Figure 4:**
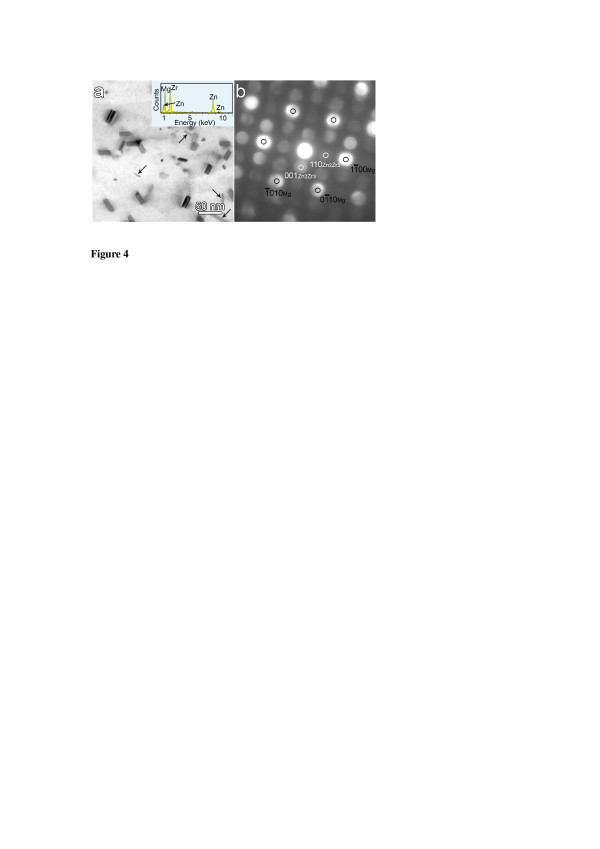
**The nanoscale Zr-rich [0001]**_**α**_**rod-like precipitates in the solution-treated alloy. (a)** [0001]_α_ TEM micrograph and the EDXS spectrum (inset), and **(b)** micro-beam electron diffraction pattern.

To explore the crystallographic characteristics of these Zr-rich [0001]_α_ rods, we examined the as-quenched microstructure using TEM with the beam parallel to the [0001]_α_ direction, as shown in Figure 4. Most of the Zr-rich particles (>80%) of the as-quenched sample in Figure 4a have a low aspect ratio in the range of 1:1 to approximately 1:3 and a thickness in the range of 6 to approximately 12 nm with their long side, which is less than 25 nm, parallel to the $<11\bar{2}0{>}_{\alpha }$ directions. They are Zr-rich [0001]_α_ rod/lath particles observed previously by STEM examinations (Figure 1a). The rest of the Zr-rich particles (<20%), marked with black arrows in Figure 4a, are thin rods with aspect ratios of 1:3 to approximately 1:20 and a thickness of 2 to approximately 5 nm, with their long axis approximately 23° away from the $<11\bar{2}0{>}_{\alpha }$ directions. They are similar to the type C Zr-rich rods reported by Gao et al [8]. In contrast, the size and aspect ratio of the dominant Zr-rich [0001]_α_ rods/laths in the end-on view are significantly different from the Zr-rich $<11\bar{2}0{>}_{\alpha }$ rods reported by Gao et al [8]. This difference is possibly due to the different alloy systems and the heat treatment techniques.

Chemical microanalysis of these [0001]_α_ rods using EDXS indicated that the atomic ratio of Mg:Zn:Zr was about 51:19:30 (inset, Figure 4a), suggesting that these [0001]_α_ rods were Zr-rich precipitates with a Zn:Zr ratio close to 2:3. The corresponding micro-beam diffraction patterns (Figure 4b) confirm that these Zr-rich [0001]_α_ rods have a tetragonal structure similar to that of Zn_2_Zr_3_ δ phase (a = b = 7.633 Å, c = 6.965 Å, α = β = γ = 90 [8,9]). The orientation relationship (OR) implied by the superimposed precipitate and matrix patterns was such that ${\left[1\bar{1}0\right]}_{\delta }//{\left[0001\right]}_{\alpha }$${\left(110\right)}_{\delta }//{\left(1\bar{1}00\right)}_{\alpha }$ and ${\left(001\right)}_{\delta }//{\left(\bar{1}\bar{1}20\right)}_{\alpha }$. By combing the commonly reported OR between β_1_′-MgZn_2_[3,10] /β_1_′-Mg_4_Zn_7_[11,12] and α-Mg matrix with the OR of the δ-Zn_2_Zr_3_ phase determined in this work, the possible ORs and the crystallographic disregistries between δ phase and β_1_′ phase were determined and listed in Table 1. The inter-planar misfits between the matching planes ${\left(001\right)}_{\delta -Zn_{2}Zr_{3}}//{\left(0001\right)}_{\beta_{1}{}^{\prime }}{}_{-MgZn_{2}}$${\left(001\right)}_{\delta -Zn_{2}Zr_{3}}//{\left(\bar{12}70\right)}_{\beta_{1}{}^{\prime }}{}_{-Mg_{4}Zn_{7}}$${\left(110\right)}_{\delta -Zn_{2}Zr_{3}}//{\left(630\right)}_{\beta_{1}{}^{\prime }}{}_{{}_{-Mg_{4}Zn_{7}}}$ and the directional misfits along the matching directions ${\left[1\bar{1}0\right]}_{\delta }{}_{-Zn_{2}Zr_{3}}//{\left[11\bar{2}0\right]}_{\beta_{1}{}^{\prime }-MgZn_{2}}$${\left[1\bar{1}0\right]}_{\delta }{}_{-Zn_{2}Zr_{3}}//{\left[001\right]}_{\beta_{1}{}^{\prime }-Mg_{4}Zn_{7}}$ were calculated as 2.5%, 5.4%, 5.1% and 3.2%, 1.8%, which are less than the critical values of 6% and 10% given in the edge-to-edge matching model [13]. The low lattice mismatch between these two phases explains why β_1_′ rods form directly on the end plane (001)_δ_ of the Zr-rich rods, as shown in Figures 2 and 3. The presence of the initial Zr-rich phases can provide much lower activation energy barrier and a favourable crystallographic correlation for the nucleation of the subsequent Zn-rich precipitates according to the classical nucleation theory [14].

**Table 1:** **Calculated misfit values between β**_**1**_**′-MgZn**_**2**_**/β**_**1**_**′-Mg**_**4**_**Zn**_**7**_**and δ-Zn**_**2**_**Zr**_**3**_**phases**

**Matching direction/plans**	**Spacing or length (nm)**	**Misfit (%)**
${\left[1\bar{1}0\right]}_{\delta }{}_{-Zn_{2}Zr_{3}}//{\left[11\bar{2}0\right]}_{\beta_{1}{}^{\prime }-MgZn_{2}}$	$L_{{\left[1\bar{1}0\right]}_{\delta -Zn_{2}Zr_{3}}}=1.0791\text{,}\;L_{{\left[11\bar{2}0\right]}_{\beta }{}_{{{}_{1}}^{\prime }-MgZn_{2}}}=0.5223$	3.2
${\left(001\right)}_{\delta -Zn_{2}Zr_{3}}//{\left(0001\right)}_{\beta_{1}{}^{\prime }}{}_{-MgZn_{2}}$ (end plane)	$d_{{\left(001\right)}_{\delta -Zn_{2}Zr_{3}}}=0.6965\text{,}\;d_{{\left(0001\right)}_{\beta }{{}_{{{}_{1}}^{\prime }}}_{-MgZn_{2}}}=0.8568$	2.5
${\left(100\right)}_{\delta -Zn_{2}Zr_{3}}//{\left(\bar{1}100\right)}_{\beta_{1}{}^{\prime }}{}_{-MgZn_{2}}$ (side plane)	$d_{{\left(110\right)}_{\delta }{}_{-Zn_{2}Zr_{3}}}=0.5397\text{,}\;d_{{\left(\bar{1}100\right)}_{\beta }{}_{{{}_{1}}^{\prime }-MgZn_{2}}}=0.4523$	16.2
${\left[1\bar{1}0\right]}_{\delta }{}_{-Zn_{2}Zr_{3}}//{\left[001\right]}_{\beta_{1}{}^{\prime }-Mg_{4}Zn_{7}}$	$L_{{\left[1\bar{1}0\right]}_{\delta -Zn_{2}Zr_{3}}}=1.0791\text{,}\;L_{{\left[001\right]}_{\beta }{}_{{{}_{1}}^{\prime }-Mg_{4}Zn_{7}}}=0.2746$	1.8
${\left(001\right)}_{\delta -Zn_{2}Zr_{3}}//{\left(\bar{12}70\right)}_{\beta_{1}{}^{\prime }}{}_{-Mg_{4}Zn_{7}}$ (end plane)	$d_{{\left(001\right)}_{\delta -Zn_{2}Zr_{3}}}=0.6965\text{,}\;d_{{\left(\bar{12}70\right)}_{\beta }{{}_{{{}_{1}}^{\prime }}}_{-Mg_{4}Zn_{7}}}=0.0549$	5.4
${\left(110\right)}_{\delta -Zn_{2}Zr_{3}}//{\left(630\right)}_{\beta_{1}{}^{\prime }}{}_{{}_{-Mg_{4}Zn_{7}}}$ (side plane)	$d_{{\left(110\right)}_{\delta }{}_{-Zn_{2}Zr_{3}}}=0.5397\text{,}\;d_{{\left(630\right)}_{\beta }{}_{{{}_{1}}^{\prime }-Mg_{4}Zn_{7}}}=0.2835$	5.1

d, spacing; L, length.

It is a significant finding that the Zr-rich phases can act as the precursor phase for the heterogeneous nucleation of Zn-rich β-type strengthening phases in the Mg alloy, given that the Zr-rich core region is a major microstructural feature of Zr-containing Mg alloys [7,8]. By effectively engineering Zr-rich [0001]_α_ rods in the Zr-rich cores of Mg alloys using a solution treatment, the formation of [0001]_α_ β_1_′ rods could be promoted according to the heterogeneous nucleation mechanism revealed by this research.

## Conclusions

In summary, we have demonstrated that the nanoscale Zr-rich [0001]_α_ rods/laths were predominant in Zr-rich core regions of the Mg-6Zn-0.5Cu-0.6Zr (wt.%) alloy after a solution treatment at 430°C. The nanoscale Zr-rich particles served as a precursor phase for the heterogeneous nucleation of the Zn-rich β-type strengthening precipitates during subsequent isothermal ageing at 180°C. These results are important for controlling Zr-rich particles in the Zr-rich core regions for enhancing the overall strength of the Mg alloy.

## Abbreviations

OR, orientation relationship; STEM, scanning transmission electron microscopy; TEM, transmission electron microscopy; HAADF, high-angle annular dark field; EDXS, energy dispersive X-ray spectrometer.

## Competing interests

The authors declare that they have no competing interests.

## Authors’ contributions

HZ conducted all the experiments and drafted the manuscript. GS, JL and CL designed the experiments and supervised the whole study. HWL and CW participated in the measurements and data analysis. RZ helped in the experiments and characterization. ZL and SPR provided the financial and technical support to the study. All the authors read and approved the final manuscript.

## References

[B1] Clark JB Transmission electron microscopy study of age hardening in a Mg-5 wt.% Zn alloy Acta Metall 1965 13 1281 10.1016/0001-6160(65)90039-8

[B2] Bettles CJ Gibson MA Venkatesan K Enhanced age-hardening behaviour in Mg–4wt.% Zn micro-alloyed with Ca Scripta Mater 2004 51 193 10.1016/j.scriptamat.2004.04.020

[B3] Mendis CL Oh-ishi K Kawamura Y Honma T Kamado S Hono K Precipitation-hardenable Mg-2.4Zn-0.1Ag-0.1Ca-0.16Zr (at.%) wrought magnesium alloy Acta Mater 2009 57 749 10.1016/j.actamat.2008.10.033

[B4] Zeng XQ Zhang Y Lu C Ding WJ Wang YX Zhu YP Precipitation behavior and mechanical properties of a Mg-Zn-Y-Zr alloy processed by thermo-mechanical treatment J Alloys Comp 2005 395 213 10.1016/j.jallcom.2004.10.070

[B5] Zhu HM Sha G Liu JW Wu CL Luo CP Liu ZW Zheng RK Ringer SP Microstructure and mechanical properties of Mg-6Zn-xCu-0.6Zr (wt.%) alloys J Alloys Comp 2011 509 3526 10.1016/j.jallcom.2010.12.165

[B6] Unsworth W New magnesium alloy for automobile applications Light Metal Age 1987 8 10

[B7] Morgan JE Mordike BL Development of creep resistant magnesium rare earth alloys 1983 Pergamon Press, Oxford

[B8] Gao X Muddle BC Nie JF Transmission electron microscopy of Zr-Zn precipitate rods in magnesium alloys containing Zr and Zn Philos Mag Lett 2009 89 33 10.1080/09500830802524096

[B9] Petersen DR Rinn HW A new phase in the zinc-zirconium system Acta Crystallogr 1961 14 328

[B10] Luo CP Liu JW Liu HW Effects of Al/Zn ratio on the microstructure and strengthening of Mg-Al-Zn alloys Mater Sci Forum 2005 488-489 205

[B11] Gao X Nie JF Characterization of strengthening precipitate phases in a Mg-Zn alloy Scripta Mater 2007 56 645 648 10.1016/j.scriptamat.2007.01.006

[B12] Singh A Tsai AP Structural characteristics of β1′ precipitates in Mg-Zn-based alloys Scripta Mater 2007 57 941 944 10.1016/j.scriptamat.2007.07.028

[B13] Zhang MX Kelly PM Edge-to-edge matching model for predicting orientation relationships and habit planes-the improvements Scripta Mater 2005 52 963 10.1016/j.scriptamat.2005.01.040

[B14] Porter DA Easterling KE Sherif MY Phase Transformations in Metals and Alloys 2009 3 CRC Press, Florida

